# Autophagy, microbiota and intestinal oncogenesis

**DOI:** 10.18632/oncotarget.5966

**Published:** 2015-10-04

**Authors:** Jonathan Lévy, Béatrice Romagnolo

**Affiliations:** Institut Cochin, INSERM U1016, Centre National de la Recherche Scientifique (CNRS), UMR8104, Université Paris Descartes, Paris, France

**Keywords:** colon cancer, autophagy, microbiota, Immune response, metabolism

Colorectal cancer (CRC) results from a series of histological changes, known as the ‘adenoma-carcinoma’ sequence, each accompanied by alterations to a specific oncogene or tumor suppressor gene. Loss of the *APC* gene has been identified as a key early event in the development of sporadic CRC, and subsequent mutations of other genes, such as *KRAS, SMAD4 and TP53,* have been shown to promote transformation ^1^. These genes are thought to drive CRC, but it remains unclear how the corresponding pathways affect cancer development. The identification of new drivers of CRC is a major challenge not only in terms of the accumulation of fundamental knowledge but also for the development of diagnostic and therapeutic tools.

We explored new molecular and cellular mechanisms, by using an *Apc* mutant mouse model of intestinal carcinogenesis and focusing on the potential role of autophagy, an essential intracellular catabolic pathway for the recycling of cellular components ^2^. There is growing evidence to suggest that autophagy plays a complex role in tumorigenesis ^3^. Autophagy has been shown to be oncosuppressive at early stages of tumor formation. However, at late stages of tumor progression, it seems to be required for the progression of adenomas to carcinomas. We found that intestinal epithelial cells (IECs) contained autophagolysosomes during tumorigenesis and that the expression of key autophagy-related genes (*Atg*) was induced throughout tumorigenesis ^4^. Similar findings were obtained for human CRC samples, 20% of which present an activation of *ATG* gene transcription. We used a conditional deletion of the *Atg7* gene to investigate the effects of inhibiting autophagy in IECs during tumorigenesis, in mice with a conditional *Apc* knockout ^5^. We found that the absence of ATG7 from IECs was associated with the development of a much smaller number and a smaller size of intestinal adenomas in *Apc*^+/−^ mice. So how does the inhibition of autophagy prevent intestinal tumor initiation and progression? We analyzed the normal mucosa of *Apc*^+/−^*Atg7*^−/−^ mice before the initiation of tumorigenesis. We found that the inhibition of autophagy in IECs induced a type I IFN-related response known to mediate antineoplastic effects against several types of cancer. This is consistent with the higher proportion of CD103^+^CD11b^−^ dendritic cells, greater production of the T-cell stimulating factor IL-12 and infiltrations with regulatory T cells and CD8^+^ T cytotoxic cells observed. Anti-CD8 antibody treatment abolished the protective antitumoral response mediated by autophagy deficiency in *Apc*^+/−^*Atg7*^−/−^ mice, whereas CD4 depletion had no such effect. We investigated the mechanism underlying the induction of Cd8^+^ T-cell infiltration by the inhibition of IEC autophagy further. The inhibition of IEC autophagy has been shown to downregulated host immunity by decreasing the antimicrobial defenses mediated by Paneth and goblet cells ^6^. We also found that autophagy inhibition changed the composition of the gut bacterial community, increasing the permeability of the intestinal barrier, with bacteria present in the crypt compartment. The gut microbiota is known to affect the intestinal immune system, and recent studies have shown it to be required for the cytotoxic response to chemotherapy ^7^. We investigated the causality of the relationship between the gut microbiota and the antitumor response mediated by autophagy deficiency, by treating *Apc*^+/−^*Atg7*^−/−^ mice with a cocktail of broad-spectrum antibiotics. Remarkably, in this context, the hypersecretion of IL-12 and the infiltration of CD8^+^ T cells were abolished, and the mice developed intestinal tumors. These results indicate the importance of the effect of the commensal bacteria on the immune system for the antitumor efficacy of autophagy inhibition.

How does the repression of autophagy inhibit adenoma growth? Here, the situation is different. We show that autophagy inhibition in tumor cells has no effect on immune cell infiltration. However, it decreases tumor growth by inducing a stress response that compromises tumor cell proliferation. Autophagy-deficient tumor cells display several types of signaling, including the activation of AMPK kinase and p53 signaling, and they have low levels of glycolytic enzymes. This suggests that the inhibition of autophagy impedes cancer development by inhibiting cancer cell metabolism.

**Figure 1:**
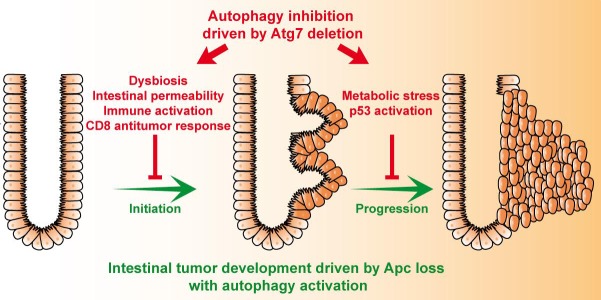
Model summarizing key aspects of the antitumoral functions of IEC autophagy inhibition in intestinal tumorigenesis driven by *Apc* loss IEC autophagy inhibition by *Atg7* deletion alters both the initiation and progression of intestinal tumorigenesis driven by *Apc* loss. At early stages, the inhibition of IEC autophagy leads to a shift in the composition of the gut microbiota, an increase in intestinal permeability and an activation of the immune response. The infiltration of cytotoxic CD8^+^ T cells has a major impact on the inhibition of tumor initiation. In addition, *Atg7* deletion in tumor cells induces a stress response characterized by the activation of AMPK and p53 and a decrease in glycolytic enzyme levels.

This newly identified anticancer function of autophagy inhibition in intestinal tumorigenesis provides opens up new possibilities for CRC treatment. Furthermore, this study highlights the importance of analyzing the role of autophagy inhibition in different types of cancers. In the pancreas and lung, autophagy inhibition results in a predisposition to premalignant lesions ^3^, whereas, in the intestine, autophagy inhibition optimizes the efficacy of cancer treatment through effects on both tumor progression and tumor initiation (Figure 1). The results of this study raise several intriguing questions. As more than half of human CRCs have *TP53* mutations, we need to determine whether tumor growth is still prevented by autophagy inhibition in cases of *p53* inactivation. In addition, our study suggests that the gut microbiota has potent antitumoral properties. Further studies are now required to understand the mechanism by which the microbiota promotes T-cell activation.
